# Population distribution models: species distributions are better modeled using biologically relevant data partitions

**DOI:** 10.1186/1472-6785-11-20

**Published:** 2011-09-19

**Authors:** Sergio C Gonzalez, J Angel Soto-Centeno, David L Reed

**Affiliations:** 1 Florida Museum of Natural History, Division of Mammals, University of Florida, Dickinson Hall, Gainesville, FL 32611, USA; 2 Department of Biology, University of Florida, Bartram-Carr Hall, Gainesville, FL 32611, USA; 3 Fort Lauderdale Research and Education Center, University of Florida, 3205 College Ave., Davie, FL 33314, USA

## Abstract

**Background:**

Predicting the geographic distribution of widespread species through modeling is problematic for several reasons including high rates of omission errors. One potential source of error for modeling widespread species is that subspecies and/or races of species are frequently pooled for analyses, which may mask biologically relevant spatial variation within the distribution of a single widespread species. We contrast a presence-only maximum entropy model for the widely distributed oldfield mouse (*Peromyscus polionotus*) that includes all available presence locations for this species, with two composite maximum entropy models. The composite models either subdivided the total species distribution into four geographic quadrants or by fifteen subspecies to capture spatially relevant variation in *P. polionotus *distributions.

**Results:**

Despite high Area Under the ROC Curve (AUC) values for all models, the composite species distribution model of *P. polionotus *generated from individual subspecies models represented the known distribution of the species much better than did the models produced by partitioning data into geographic quadrants or modeling the whole species as a single unit.

**Conclusions:**

Because the AUC values failed to describe the differences in the predictability of the three modeling strategies, we suggest using omission curves in addition to AUC values to assess model performance. Dividing the data of a widespread species into biologically relevant partitions greatly increased the performance of our distribution model; therefore, this approach may prove to be quite practical and informative for a wide range of modeling applications.

## Background

Species distribution modeling (SDM) has become a common tool for understanding spatial distribution patterns of biodiversity worldwide [1-4]. The goal of SDM is to build a model predicting the relative probability of occurrence of a species across geographic space commonly using environmental data (i.e. climate, vegetation, soil, etc.) and a dataset of known presence or presence/absence localities. The terms *ecological niche model, environmental niche model*, and *species distribution model *have all been used to describe this type of modeling in the literature; for the sake of simplicity we will use species distribution modeling. SDM techniques continue to evolve with an increasingly broad range of applications from conservation planning [5,6], to predicting species colonization and abundance [4,7,8], predicting disease outbreaks [1], and understanding phylogeographic patterns [9]. Methods of producing SDMs vary with the type of data available, purpose, and software used.

There is a direct link between climate and the distribution of plant species [10]. Because climate is a causal factor in the distribution of plant species (and plant species assemblages), climatic patterns at various spatial scales directly affect habitat types and community productivity. Thus, climate is considered a proxy for a given species' environmental niche. Because of the variables involved in building SDMs, it is important to keep in mind that SDMs are predicting a species' fundamental niche as probability of occurrence [11,12], not the realized distribution, which is affected by many extrinsic factors that may not be accounted for in the model. The fundamental niches of species are considered to be conserved over evolutionary time [11], which has allowed climate-based SDMs to be successful in predicting the occurrence of species or closely related species at previously unsampled localities [13-16].

Modeling species whose distributions span large environmental or habitat variation may be problematic because distribution models tend to have higher rates of omission error (i.e. underprediction) in the predicted species distributions [2,17,18]. In such cases, the models may indicate regional specialization of periphery or isolated populations. Commission errors (i.e. the overprediction of distributions) may result from a restriction of the realized distribution due to biotic interactions or geographic barriers to range expansion. In an attempt to overcome omission errors in SDMs for widespread species, Osborne & Suarez-Seoane [17] modeled species distributions by spatially partitioning their data into geographic quadrants and into concentric rings to model each data partition separately. Hernandez et al. [18] suggested that future research should focus on modeling broad distributions in subunits that are based on distinct genetic lineages or recognized subspecies.

Most species in the genus *Peromyscus *are widespread, with a positive correlation between species range and number of recognized subspecies [19], suggesting that local specialization is common within species of this genus. With 15 recognized subspecies [20] and genetically structured populations, *P. polionotus *is an excellent model species for developing new methods of data partitioning to overcome the problems associated with modeling the geographic distributions of widely distributed species.

**Figure 1:**
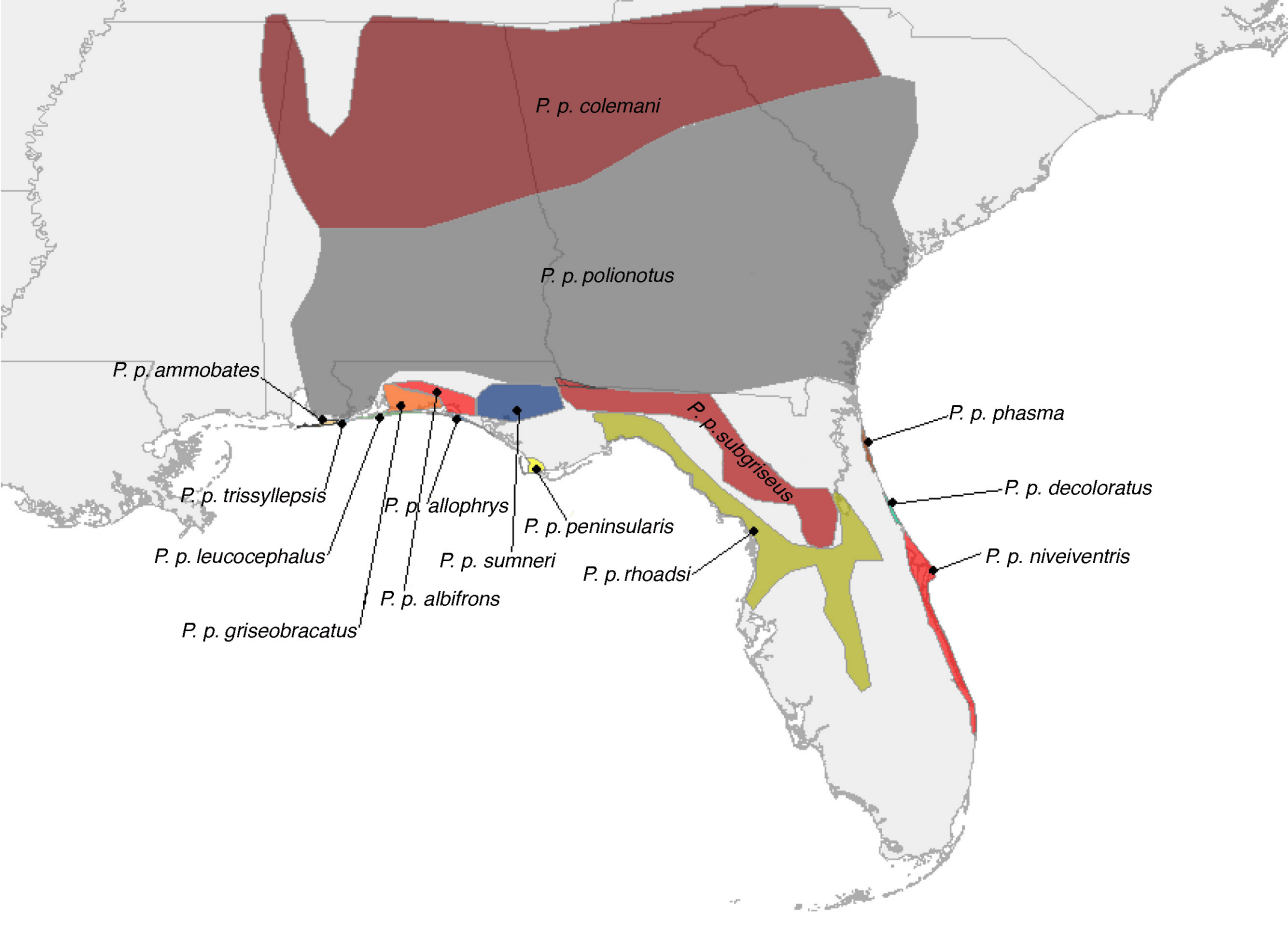
***P. polionotus in southeastern United States***. Distribution map of the 15 supspecies of *P. polionotus *redrawn from Hall (1981).

The oldfield mouse (*Peromyscus polionotus*), also known regionally as the Florida beach mouse, is widespread throughout the southeastern United States (Figure 1). Morphological and genetic differences have been documented between subspecies [21-25]. Molecular evidence suggests that little or no gene flow occurs between the highly structured populations on islands along Florida's panhandle [25] or between *P. p. rhoadsi*, on Florida's central ridge, and *P. p. niveiventris*, on the Atlantic coast [21]. Highly differentiated populations of *Peromyscus *are suggested to be the result of strong local adaptation [19,23,26]. Coastal dune populations along the Gulf of Mexico are phenotypically more similar to populations along the Atlantic Coast (especially in coat color) than to neighboring populations, suggesting they are under similar selective pressures in their disjunct coastal environments [23]. Throughout its range, *P. polionotus *is threatened by development and invasive species exerting competition and predation pressures on its populations, and the subspecies *P. p. decoloratus *has been considered extinct since 1950 [19,26].

Given the evidence for local adaptation in this widespread species, previous research would predict that an SDM of the entire species would underpredict the geographic distribution of *P. polionotus*. A more accurate prediction of the species' distribution may result from a biologically informed spatial partitioning of locality data. To test this hypothesis, we modeled the distribution of *P*. *polionotus *in three ways; we modeled the whole species distribution at once, we partitioned locality data into four geographic quadrants following Osborne & Suarez-Seoane [17], and we partitioned locality data by the 15 recognized subspecies of *P. polionotus*.

## Results

**Figure 2:**
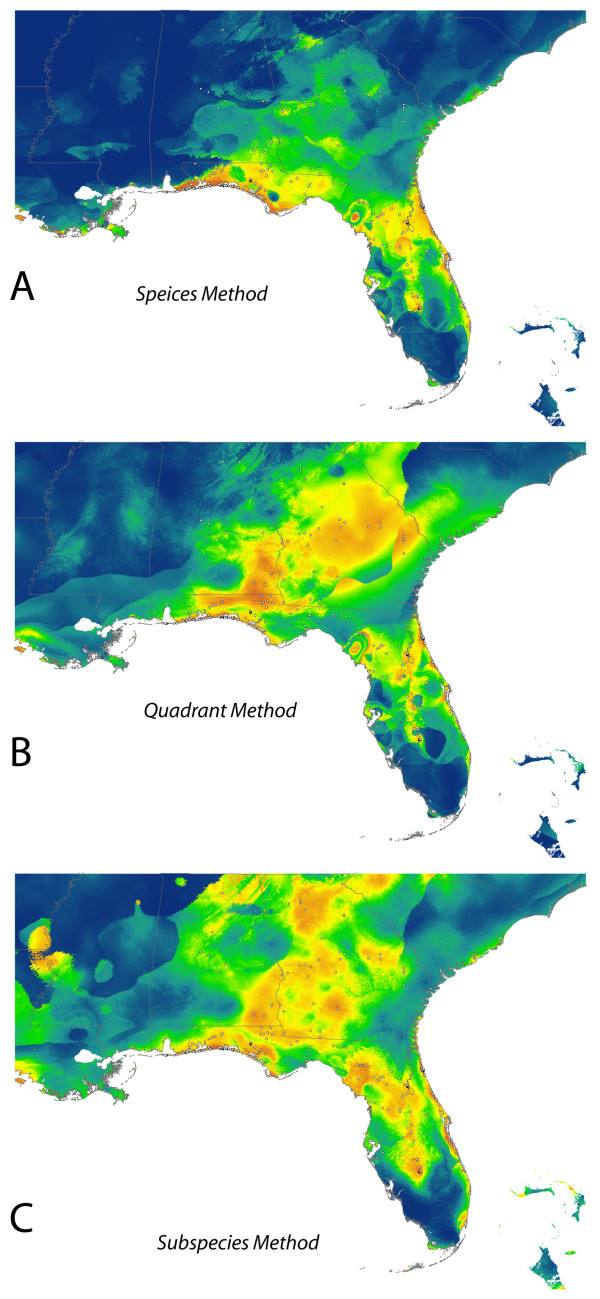
**Maxent distributions for *P. polionotus *using different data partitions**. Predicted species distributions using presence data for the entire species at once (A), by dividing the presence data into quadrants (B), and by subspecies (C).

All individual and composite models produced AUC values above 0.84, which are consistent with AUC values reported in the literature for other taxa [9,17,18,27]. Despite having a high AUC value (0.899), the model based on the entire data set failed to predict the occurrence of *P. polionnotus *in places where it is clearly known to occur (Figure 2a), most obviously omitting the distributions of the subspecies *P. p. colemani *and *P. p. polionotus*. Partitioning the data by geographic quadrants (Figure 2b) and by subspecies (Figure 2c) produced models that are progressively better, both in terms of predicting the known distribution, and in terms of their AUC scores. The average AUC value of the individual models used to build the geographic quadrant composite model was 0.927, whereas the average AUC value of the individual models that were used to build the subspecies composite model was 0.976.

**Figure 3:**
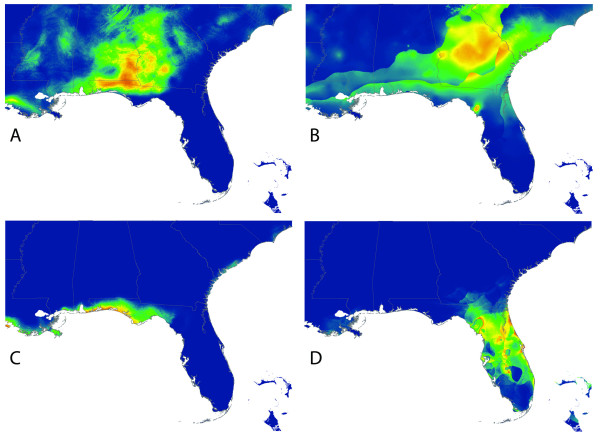
**Maxent distribution based on the quadrant method**. Predicted species distribution of *P. polionotus *estimated from presence data modeled separately in four quadrants (A-D).

**Figure 4:**
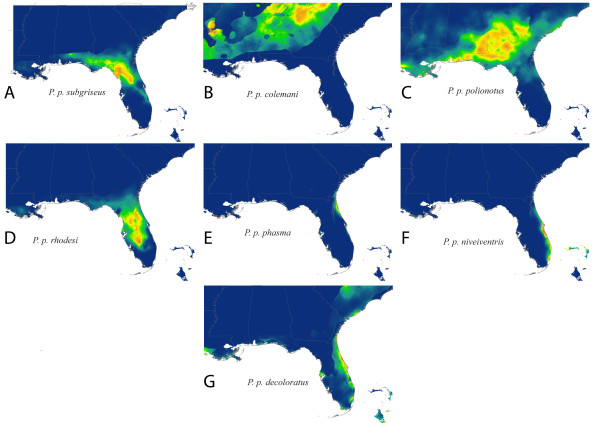
**Maxent distribution of *P. polionotus *on peninsular Florida**. Predicted species distribution of *P. polionotus *estimated from presence data of each mainland and peninsular subspecies (A-G) analyzed separately.

**Figure 5:**
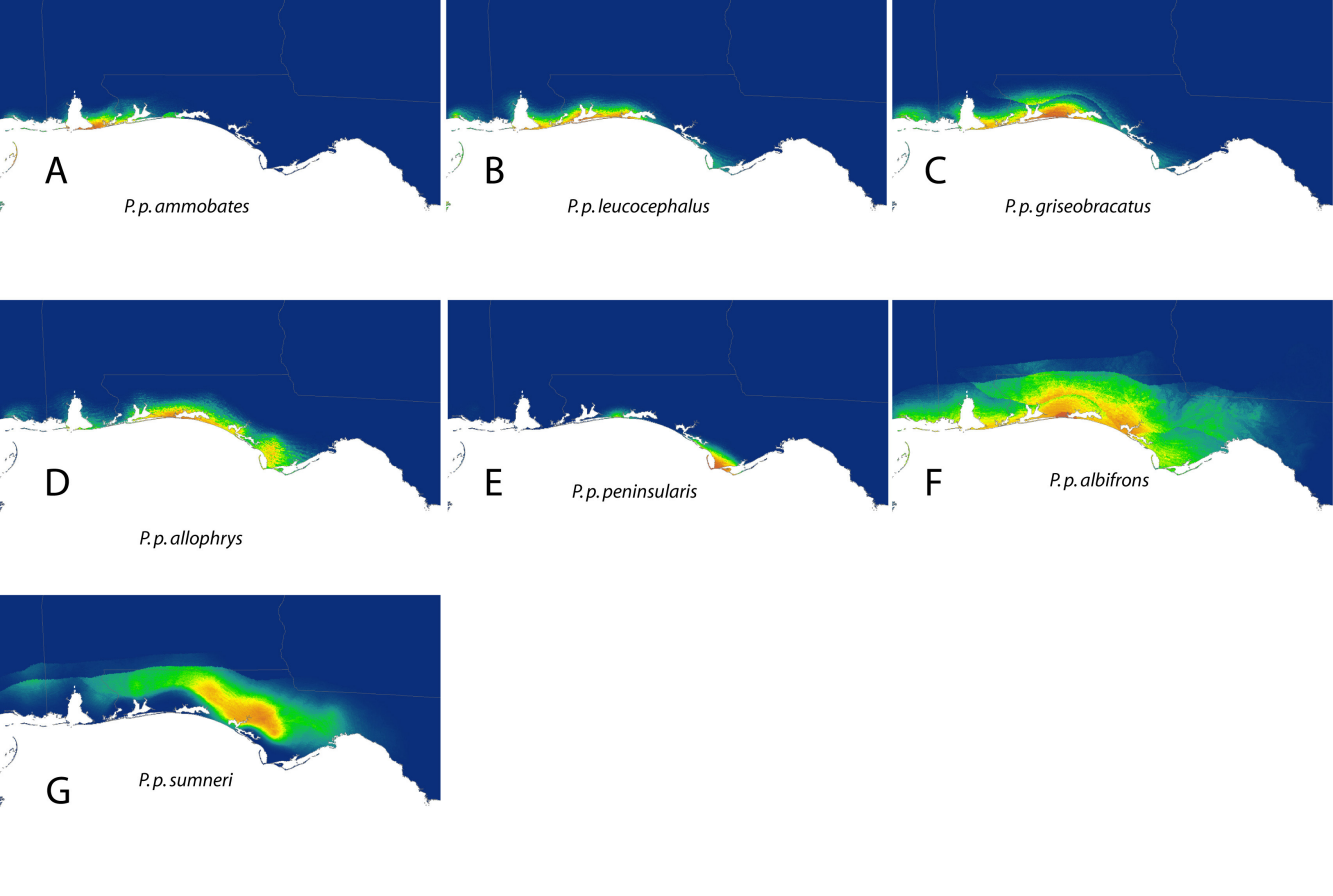
**Maxent distribution of *P. polionotus *on Florida panhandle**. Predicted species distribution of *P. polionotus *estimated from presence data of each Florida panhandle subspecies (A-G) analyzed separately.

Partitioning the data into geographic quadrants produced four models with AUC scores of 0.844, 0.968, 0.905, and 0.993 (clockwise from northeast; Figure 3). The quadrant composite model (Figure 2b) predicted high probabilities of occurrence in areas that the full species model (Figure 2a) had omitted. However, the quadrant composite model (Figure 2b) showed poor resolution in parts of northern Georgia and peninsular Florida. When the dataset was partitioned according to the currently recognized subspecies (Figures 4 and 5), each subspecies model performed well based on AUC scores. Twelve out of 15 models had AUC scores between 0.97 and 1.0; the exceptions being *P. p. colemani *(0.917), *P. p. polionotus *(0.851), and *P. p. trissyllepsis *(0.5). The poor performance of the model for *P. p. trissyllepsis *was due to insufficient data (*n *= 2) for the population and was omitted from the composite model.

**Figure 6:**
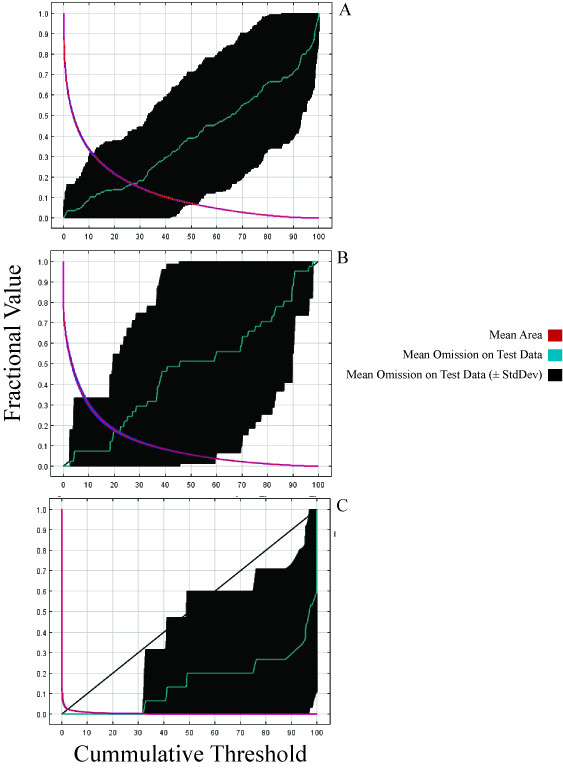
**Omission curves as a means of assessing model performance**. Omission curve (thin green line) for the species level model of *P. polionotus *(A), the northwest region of the quadrant method (B), and the single subspecies *P. p. phasma *of the subspecies method (C). Mean AUC value for replicate runs was 0.899 (std.dev. = 0.105), 0.905 (std.dev. = 0.119), and 1.0 (std.dev. = 0.001), respectively.

Although superficially all models developed from data partitions seem to perform well based on AUC values, rates of omission between these methods show a different perspective. Figure 6 shows three omission curves for the whole-species model (Figure 6a), the quadrant method (Figure 6b) and the subspecies model (Figure 6c). The curves show omission error (Y-axis) as a function of predicted probability of occurrence (X-axis). Better performing models based on the logistic output of Maxent have fewer omission errors even as predicted probability of occurrence reaches maximal values. The whole-species model (Figure 6a) and the quadrant model (Figure 6b) have omission error rates that increase linearly with increasing probability of occurrence. In contrast, the subspecies model (Figure 6c) has relatively low omission error rates that only begins to increase when predicted probability of occurrence reaches higher values, which is preferable.

## Discussion

We suggest that partitioning data in a biologically meaningful way (as opposed to geographically) can help to overcome high omission rates in distribution models of widespread species [17,18,28]. Despite having a relatively high AUC value (0.899), the model built upon the whole species' distribution failed to predict known localities of *P. polionotus*, which can be observed by comparing Figure 1 to Figure 2a. Spatially partitioning the data into quadrants produced a much better distribution model after combining four regional models with AUC values ranging from 0.844 to 0.993, which can be seen by comparing Figure 1 to Figure 2b. The 14 AUC scores for the SDMs based on subspecies partitioning ranged from 0.851 to 1.0, which is not substantially different from AUC values obtained for the quadrant models. However, the accuracy and increased resolution of the composite of the subspecies models can be seen in comparing the three panels of Figure 2 and by comparing Figure 1 to Figure 2c.

### Limitation of AUC values for assessing predictive performance

AUC values are commonly used as indicators of model fit [9,17,18,27], and high values for all three methods in our study would suggest that each method produced highly accurate models. Furthermore, the modest increase in AUC values with ever greater data partitioning would suggest that each successive partitioning scheme produced, at best, only slightly better fitting models. However, this finding is misleading when one compares the predicted distributions to the known distribution for the species. The trend of increasing AUC scores may indicate the direction of change in accuracy, but it fails to capture the magnitude of improvement in the predicted distributions of the quadrant composite method and the subspecies composite method. This failure is, in part, due to the fact that the AUC scores for the geographic quadrant method and subspecies method are composites of 4 and 14 combined models (respectively), and the accuracy (or inaccuracy) of individual models is compounded when they are combined. This fact alone, however, cannot fully explain the observed discrepancy between the vastly improved model prediction and the modestly better AUC scores.

AUC values can be misleading when assessing a model's predictive ability for several reasons. The AUC measures discrimination and not accuracy *per se*, thus ignoring the goodness of fit of a model [29]. The AUC value also takes into account the performance of the model at the extreme left (as well as the right) of the ROC curve (see [29] for a details), a region that is not operationally meaningful in our case. We are only interested in thresholds of predicted probability of occurrence greater than 0.50 because that would equal the probability of occurrence of a null model. This inclusion of the area under the extreme left of the ROC curve can inflate AUC values, which can be further inflated when the total geographic extent of the model is considered. If the ratio between areas of presence and the total extent is high, true positives are more likely to occur by chance alone [28]. Because this ratio changes with each of the individual models built on different data partitions, AUC values may not be useful in accurately comparing relative model performance between or among our subspecies and regional models.

It is also possible that the inflation of AUC values observed in the models we present results from the interaction between geographic and environmental space. Because of the narrow geographic space at which most subspecies of *P. polionotus *occur, locality information is geographically clustrered; therefore, the environmental space sampled by the models may show spatial autocorrelation in some of the environmental variables used. In SDM, spatial autocorrelation occurs when the values of the variables sampled at nearby locations are not independent from each other [30] and as a result, measures of accuracy (e.g. AUC) can be inflated [31,32]. In our case, the geographic clustering of narrowly distributed subspecies of *P. polionotus *may cause spatial autocorrelation and thus inflate AUC values (see Figure 4 and 5). Nevertheless, within these narrow extents, we included samples spanning the entire geographic space representing a significant portion of the environmental space occupied by each subspecies. The resulting models are accurate to the true distribution of the subspecies and are able to detect even subtle local environmental conditions likely affecting each subspecies differently, despite the seemingly geographic clustering. This further emphasizes the point that AUC values provide an unreliable way to accurately compare relative model performance. The only exceptions to the issue of inflated AUC values in our dataset are *P. p. colemani *(AUC = 0.917) and *P. p. polionotus *(AUC = 0.851), which are the only two widely distributed subspecies spanning a more heterogeneous environmental space where locality information for the subspecies is not geographically clustered (Figure 2). Because of the larger geographic space occupied by these subspecies, the resulting models from *P. p. colemani *and *P. p. polionotus *are unlikely affected by spatial autocorrelation and therefore do not show inflated AUC values.

Finally, the AUC does not provide information as to the spatial distribution of errors. It also weighs omission and commission errors equally, both of which vary in interpretive meaning and importance with the intended use of the model [29]. Because we do not have true absence data, we cannot quantify our commission error rate. However, the omission curve shows how well the model performs at different thresholds (i.e. the distribution of omission errors). Therefore, the omission curve can be as important as the AUC value in terms of assessing model performance, if not more-so. A model with relatively lower omission errors at higher predicted probabilities of occurrence is preferred.

### Quadrant versus subspecies partitioning

Because Maxent draws pseudo-absence data at random to calculate AUC scores, it is possible that in our quadrant analysis it drew false pseudo-absences from areas outside the quadrant being tested, especially in the case of the northwest quadrant. Whether this occurred, and if it did, whether it contributed to the observed underprediction is debatable. First, there was far less underprediction in the other three quadrants. For example, the model for the southwest quadrant, which includes peninsular Florida predicted occurrences in regions where the species does not occur. Second, the two northern quadrants cover roughly the same geographic area as two of the subspecies (*P. p. colemani *and *P. p. polionotus*), yet the two northern quadrants failed to predict areas of known occurrence that the two subspecies models predicted accurately. It is possible that poor quadrant-based models resulted because pseudo-absences were being generated by Maxent in areas with true presences for either of the two subspecies just outside of the quadrant being modeled. That is, the arbitrary boundary between the quadrants obfuscates biologically meaningful boundaries between populations or subspecies. Thus, in using quadrant-based partitioning, niche information was lost for the two subspecies, which emphasizes our point that biologically relevant data partitioning informs species distribution models.

### Molecular data and population distribution models

It is easy to see how molecular data, such as DNA sequences, can be used to delineate biologically meaningful groups (i.e. clades) within a species, and that those clades might be partitioned separately for species distribution modeling, especially if they are geographically discrete. But, just as molecular data can improve methods of generating SDMs, the findings associated with SDMs can also inform the work done by molecular biologists studying population genetics, phylogenetics, or phylogeography. When SDMs are nonoverlapping for populations within a species, they may be revealing cryptic patterns of divergence that would be interesting to study with molecular data. Conversely, when molecular data uncover population structure or limits to gene flow, SDMs can be used to test hypothesized mechanisms of divergence such as niche differentiation. Examining both molecular data and SDMs together has been explored only recently [6,9].

Building SDMs for *P. polionotus *by partitioning data into subspecies and building a composite distribution model mitigated the problem of high omission rates that usually occurs when modeling the distributions of widely distributed species. This suggests that the SDM based on biologically relevant partitions (subspecies in our case) could accommodate variability in the niches of subspecies, whereas modeling the whole species distribution together could not. This is supported by the fact that spatial partitioning of data into quadrants produced models that had regions of both under- and over-prediction (Figure 3), whereas the models based on partitioning by subspecies showed no signs of underprediction and only modest overlap in the distributions of adjacent individual subspecies caused by overprediction (Figure 5). The evidence of high levels of population structure between locally adapted populations [19,21-23,25,26] might be driving the improvement we see in the composite model based on subspecies distributions. In our case, we show that molecular data at the population level improved model accuracy. Furthermore, in the absence of detailed molecular information on the populations studied, researchers could generate relevant data partitions using alternative data sources such as subspecies delimitations, morophological differences or other phenotypic traits.

### Phylogeographic implications for *P. polionotus*

Guisan & Zimmermann [28] encourage collaboration with evolutionary biologists and population geneticists in cases where widespread species are being modeled. More recently, Rödder et al. [9] discussed how a variety of techniques including molecular ecology and environmental niche modeling can be complimentary in answering phylogeographic questions. The case is such here, where our method of partitioning data was based largely on the literature, which includes population genetic studies that have been conducted on *P. polionotus*. Conversely, as molecular work helped to inform our models, our models also shed light on and confirm results of studies on the species' genetic structure, and possibly evolutionary trajectory. For example, the SDMs for *P. p. rhoadsi *and *P. p. nivieventris *do not overlap and are geographically discrete (Figure 4 and 2c), which is consistent with the genetic results of Degner et al. [21] and current taxonomy (Figure 1). The southeastern quadrant model, however, has very poor resolution of this finescale distinction (Figure 3c). Similarly, the models for *P. p. polionotus *and *P. p. colemani *capture the known extent of their respective ranges (Figure 5), while the two northern quadrant models do not (Figure 3, a and 3b), implying that these two subspecies occupy different climatic niches.

Climate appears to play an important role in defining inland and inland vs. coastal subspecies (e.g. Figure 4A-D). However, despite there being genetic differences in the beach mouse subspecies located in the Florida panhandle (Figure 5A-F), there was considerable overlap in their predicted distributions, suggesting that climate may not be the primary factor defining the range of these subspecies. Predicted niche overlap usually occurred between adjacent coastal subspecies (Figure 5A-F) and only once between coastal and inland subspecies (see *P. p. albifrons *and *P. p. sumneri*, Figure 5F-G). Studies have shown that the coastal beach mouse populations reflect patterns of local adaptation and strong selection favoring cryptic coloration [22-25]. Therefore, in cases where climatic habitat on adjacent coastal beaches might be similar, vicariance (i.e. coastal inlets) and strong selection for coat coloration are more likely than climate to maintain the distinctiveness of coastal beach mouse subspecies.

## Conclusions

Using a biologically meaningful method of partitioning the data from widely distributed species generated a composite SDM of *P. polionotus *that more accurately reflected the known distribution of the species than the process of analyzing the whole species at once or partitioning the data into geographic quadrants. Osborne and Suarez-Seoane [17] note that geographic based data partitioning (e.g. quadrants) may not have worked well due to the absence of any biological basis for partitioning. We contend that our study confirms that statement. We also provide an example of how SDMs can be both informed by as well as inform phylogeographic studies at the population and species levels. Modeling a widespread species using biologically meaningful data partitions has the potential to greatly increase the performance of distribution models while only requiring basic manipulation in GIS software. Thus, this technique may prove to be quite practical for a wide range of modeling applications. Despite the increasing use and popularity of ENMs, a completely objective, accurate, and fully accepted measure of performance of predictive distribution models is still elusive [9,27,29]. We suggest using both the AUC and omission curve on a contextual basis to assess model performance.

## Methods

We created species distribution models for *P. polionotus *in Maxent using the WorldClim climate layers. Maxent uses the principle of maximum entropy density estimation to generate a probability distribution based on presence-only data [33,34]. It has been shown to produce more accurate models with lower sample sizes than other distribution modeling software [18,35]. We used the WorldClim Current BioClim climate layers at 30 arc-seconds resolution (about 1 km^2^). These layers are based on data from 1950-2000 and comprise 19 bioclimatic variables representing annual trends, seasonality, and extremes of precipitation and temperature [36]. We used the entire set of 19 climatic variables because we did not make any *a priori *assumptions of correlation among these variables. We clipped the WorldClim layers in ESRI ArcGIS 9.3 to include the extent of the species geographic range in our models (N35.00, E-77.0, W-92.0, S25.00).

Presence data was obtained from collection localities of museum specimens of *P. polionotus *identified to subspecies (Louisiana State Museum of Natural Science, Michigan State University Museum, National Museum of Vertebrate Zoology, American Museum of Natural History, University of Michigan Museum of Zoology, University of Kansas Biodiversity Institute, Sam Noble Oklahoma Museum of Natural History), found on the online data bases Mammal Network Information System [37] and Global Biodiversity Information Facility [38]. Records lacking GPS coordinates, but with specific written locality information were georeferenced following MaNIS protocols using Google Earth and the U.S. Board of Geographic Names' (BGN) Geographic Names Information System (GNIS) [39].

To obtain a model of distribution for the entire species, a model representing the mean distribution was produced in Maxent using a cross-validation approach of all specimen localities. The cross-validation function split the data set into *n *samples. In each of the *n *replicates, a single specimen was tested sequentially against all remaining samples (i.e. *n - *1), which formed the training set of localities [40]. This eliminated the need to partition a dataset into large training and testing sets. This approach is useful when dealing with especially small datasets, where splitting the data would result in a training set of insufficient size.

Similar to Osborne and Suarez-Seoane [17], we spatially partitioned our data into geographic quadrants (northeast, southeast, northwest, southwest) based on the unweighted centroid of our dataset. Using the same methods as described above, we ran models for each of the four data partitions. We note that we only partitioned our presence data. Therefore, in this case, the pseudo-absences drawn by Maxent are drawn from our complete working extent (not merely the quadrant being examined in isolation). These four models were then combined to produce a composite model of probability of occurrence for the entire species. This was done in ESRI ArcGIS using the Spatial Analyst toolbox to create a new raster based on the four independently modeled quadrants. When two or more quadrants predicted occurrence at a single point, we used the higher probability of occurrence value in our composite species distribution.

We produced a second composite model by partitioning our presence data into the 15 recognized subspecies of *P. polionotus *and modeling the distributions of each subspecies separately. The subspecies *P. p. trissyllepsis *lacked sufficient data to build a functioning model, so that subspecies was omitted. The remaining 14 subspecies models where combined in ArcGIS, as described above, to produce a composite model of probability of occurrence for the entire species.

The final logistic outputs of each model were used to assess our results. The area under the curve (AUC) of receiver operating characteristic (ROC) plot was used to evaluate model performance. The AUC is a threshold independent measure of model performance, where an AUC value of 1 indicates optimal performance, and AUC = 0.5 indicates a model performing no better than a randomly generated one. The mean and range of the AUC values of each group of models used in the composites were compared in an attempt to give a relative value of "goodness" for the two composites.

## Authors' contributions

SCG, JASC, and DLR conceived and designed the experiment. SCG and JASC carried out data analysis. SCG, JASC, and DLR designed and wrote the manuscript. All authors read and approved the final manucript.
